# Possible Benefits of a Low Protein Diet in Older Patients With CKD at Risk of Malnutrition: A Pilot Randomized Controlled Trial

**DOI:** 10.3389/fnut.2021.782499

**Published:** 2022-01-26

**Authors:** Lara Caldiroli, Simone Vettoretti, Silvia Armelloni, Deborah Mattinzoli, Masami Ikehata, Paolo Molinari, Carlo Alfieri, Piergiorgio Messa, Giuseppe Castellano

**Affiliations:** ^1^Unit of Nephrology, Dialysis and Renal Transplantation - Fondazione Istituto di Ricerca e Cura a Carattere Scientifico (IRCCS) Ca'Granda Ospedale Maggiore Policlinico di Milano, Milan, Italy; ^2^Department of Clinical Sciences and Community Health, University of Milan, Milan, Italy

**Keywords:** malnutrition, chronic kidney disease, low protein diet, FGF23, inflammation

## Abstract

**Background:**

Current guidelines do not clarify whether older patients with advanced chronic kidney disease (CKD) may benefit of low protein (LP) diet if they are at risk of malnutrition. We compared the effects of normocalorie/normoprotein (NP) and normocalorie/LP diet on nutritional status and metabolic complications related to the progression of kidney damage in these patients.

**Methods:**

This pilot study had an open-label randomized-controlled design (ClinicalTrials.gov Id: NCT05015647). Thirty-five patients were treated for 6 months with two different diets (LP = 17) and (NP = 18). Malnutrition was assessed by the Malnutrition Inflammation Score and International Society of Renal Nutrition and Metabolism criteria. Renal function was assessed by creatinine and cystatin-C-based estimated glomerular filtration rate (eGFR).

**Results:**

At the end of the study, Malnutrition Inflammation Score was improved in both LP and NP groups (respectively: 3 ± 3 vs. 6 ± 1.5, *p* = 0.020 and 3 ± 2.5 vs. 6 ± 2, *p* = 0.012), prevalence of protein energy wasting syndrome decreased only in LP. LP group had higher eGFRcys-C (17 ± 6 vs. 12 ± 4 ml/min/1.73 m^2^; *p* < 0.05), lower serum urea (105 ± 65 vs. 138 ± 30 mg/dl; *p* < 0.05) and lower parathormone (68 ± 10 vs. 99 ± 61 ng/L; *p* < 0.05) than NP. Serum and urinary phosphorous did not change while fibroblast growth factor 23 (FGF23)-intact and FGF23 c-terminal increased in both groups [FGF23-intact in LP: 70 (48; 98) vs. 126 (90; 410) pg/ml, *p* < 0.01 and in NP: 86 (57; 194) vs. 143 (119; 186) pg/ml, *p* < 0.01; FGF23 c-terminal in LP: 77 (30.3; 112) vs. 111 (63; 384) RU/ml, *p* < 0.01 and in NP: 142 (56.6; 175) vs. 157 (76.7; 281) RU/ml, *p* < 0.01].

**Conclusions:**

LP diet has a favorable impact on nutritional status as much as NP diet with possible greater benefits on the progression of kidney disease and some of its metabolic complications.

**Clinical Trial Registration:**

https://clinicaltrials.gov/ct2/show/NCT05015647, identifier: NCT05015647.

## Introduction

A significant proportion of patients (20–45%) with chronic kidney disease (CKD) spontaneously reduces its protein-calorie intake as glomerular filtration rate (GFR) decreases (1). Alterations caused by CKD reduce appetite and compromise nutritional status, leading to sarcopenia and malnutrition (2, 3).

Anorexia associated with CKD may be due to some variations of the neuroendocrine pathways that operate principally in the hypothalamus (4). Indeed, in advanced stages of CKD some substances, such as hormones (insulin, leptin, PYY3-36 produced by the colon, and ghrelin) and uremic toxins (cresols, indoles, and phenols) accumulate. These substances could reduce appetite by activating melanocortin receptor 4 (MC4-R) and by suppressing the activity of AMP-activated protein kinase (AMPK) (5).

Other conditions that can cause anorexia are changes in taste, uremic halitosis, uremic gastritis and the high number of drugs that are prescribed to these patients (5). It must be noted, however, that in studies where patients were followed by nephrologists and dietitians to mitigate the metabolic disturbances coming from the loss of kidney function, there was not an onset of protein energy wasting syndrome (6, 7).

The equilibrium of nitrogen balance is fundamental to preserve good nutritional status and body composition (5). In the stable patient with CKD, in case of reduced protein intake, nitrogen balance is maintained thanks to the adaptive capability to reduce protein catabolism, provided that calorie intake is maintained (8–10). However, if we induce a simultaneous reduction of protein and calorie intake this provokes protein degradation for energy purposes. Therefore, in patients following low protein (LP) diets, an appropriated calorie consumption is crucial to prevent progressive protein wasting (11). In non-dialysis patients with different levels of calorie intake (45, 35, 25, or 15 kcal/kg/24 h) but with LP consumption (from 0.55 to 0.60 g/kg/day), nitrogen balance correlated with calorie intake but it did not correlate with the dietary protein consumption (11). In a recent study, Garibotto et al. demonstrated that in patients with CKD subjected to LP diet (0.55 g/kg/24 h) muscle protein synthesis was unaffected provided that sufficient calorie intake was maintained. As a matter of fact, skeletal muscle responded to LP intake through the combined effects of reduced protein degradation, unchanged protein synthesis and overall increased efficiency of protein metabolism. Moreover, they observed that muscle protein turnover after a LP diet was more efficient than during normal protein intake (12). Therefore, these Authors conclude that a crucial aspect of nutrient intake in CKD subjected to LP diet is the amount of energy supply. Last KDOQI Clinical Practice Guidelines for Nutrition in CKD reported that once the provision of an adequate energy supply is assured, protein intake can be safely decreased to 0.55–0.6 g/kg/day (13).

Current guidelines (12, 13) indicate to avoid the prescription of LP diets in malnourished patients with CKD; however, the clinical characteristics defining malnutrition in patients with CKD are not universally recognized. Moreover, the guidelines do not consider which nutritional intervention should be prescribed to patients with CKD with spontaneous low energy and protein consumption that are deemed to be at risk of malnutrition.

Optimal nutritional care for elderly patient with advanced CKD at risk of malnutrition is still uncertain and there is an urgent need of evidence-based indications regarding prevention and management of malnutrition in this setting (16). In a recent review, Deer et al., suggested that a diet with a protein intake of 0.8 g/kg/day is encouraged in stable nephropathic patient given the risk of CKD progression with a high-protein diet (12–17). Only during acute phases of illness, it is recommended to increase protein intake to 1 g/kg/day (17). Moreover, Fois et al. demonstrated that protein restriction is feasible in maintaining a stable nutritional status in an elderly, high-comorbidity population (7).

In patients with advanced CKD, an increase of dietary protein intake could be associated with an increase of serum urea and phosphorous as well as a with a reduction of bicarbonate levels (6). All these variations significantly contribute to the overall metabolic derangement characterizing the uremic syndrome.

Starting from these premises the aim of this pilot study was to assess whether in older patients with CKD at risk of malnutrition a LP diet could be as safe as a normoprotein (NP) diet. In particular, our first aim was to assess whether there were differences between LP and NP diet regarding the development of malnutrition. Furthermore, we explored the effects of these different diets on the progression of kidney damage as well as on metabolic disorders associated with CKD.

## Materials and Methods

### Study Design and Characteristics of Patients

This pilot study has an open-label randomized-controlled design. Estimated number of patients should have been 38, taking in account of a maximal drop out up to 20% of the sample. We enrolled 35 patients, 27 of whom terminated the study as per protocol (14 in the LP group and 13 in the NP group). Causes of withdrawal are reported in Table 1.

**Table 1:** Population characteristics.

**Variables**	**Overall cohort** **(*n* = 35)**	**NP BL** **(*n* = 18)**	**LP BL** **(*n* = 17)**	***p***
Age (years)	81 ± 6	82 ± 6	79 ± 5	0.2
Males, *n* (%)	23 (66%)	10 (55%)	13 (77%)	0.19
Weight (Kg)	73.5 ± 14.6	73.5 ± 14.5	73.5 ± 15	0.98
BMI (Kg/m^2^)	27.3 ± 6.5	27.8 ± 6.6	26.3 ± 6.6	0.95
Diabetes, *n* (%)	14 (40%)	8 (44%)	6 (35%)	0.58
Hypertension, *n* (%)	33 (94%)	16 (89%)	17 (100%)	0.16
Previous CV events, *n* (%)	16 (46%)	9 (50%)	7 (41%)	0.6
Causes of dropouts	3	1	2	0.51
Start of dialysis, *n*				
Deaths, *n*	2	2	0	0.16
Hospitalizations, *n*	3	1	2	0.68

*BMI, body mass index; CV, cardiovascular; MIS, Malnutrition inflammation score*.

The pilot sample size was calculated using a one-sided 80% CI, with a standard deviation of 0.3 and significance of 5%. Thirty-two patients (~9% of the main sample size) is the sample size required for the pilot study to achieve a one-sided 80% confidence limit (18). The power calculation of the study was: 0.52 = 7.9 × 2.32 × (1/n + 1/n); *n* = (7.9 × 2.32 × 2)/0.52 = 334; Therefore, 334 was the number of the main sample size; we took 9% of 334, i.e., 30 that we decided to approximate at 32. Finally, we added 20% of estimated drop out, so 38 patients were the target for the pilot study. For randomization we tossed a coin (heads–LP group, tails–NP group) (19).

Inclusion criteria were: advanced CKD not yet on renal replacement therapy (10< estimated glomerular filtration rate [eGFR] creat <30 ml/min) and older age (>65 years). Furthermore, all patients had to be classified at risk of malnutrition at Malnutrition Inflammation Score (4 ≤ MIS ≤ 7) and to have spontaneous LP-energy intake (proteins <0.8 g/kg and energy <25 kcal/kg).

Exclusion criteria were: active cancer; symptomatic infectious disease in the previous 2 months; decompensated chronic liver diseases; symptomatic heart failure (NHYA II-IV); endocrine disease; intestinal malabsorption, hospitalization in the last 2 month and inability to cooperate. We also excluded all patients that were under treatment with immunosuppressive drugs and with a presumed overall life expectancy <6 months.

Patients were treated for 6 months with two different dietary prescriptions:

- LP group (*n* = 17) was prescribed normocalories/LP diet (30 kcal/kg and 0.6–0.7 g/kg, respectively). To assure prescribed calorie intake, this group was supplemented with commercial protein free products (protein content <2%).
- NP group (*n* = 18) was prescribed normocalories/normal proteins diet (30 kcal/kg and 0.8 g/kg, respectively).

All patients received at every visit a counseling by a trained nutritionist. Both groups were educated to avoid “hidden” phosphorus from additives in preserved/processed foods and to consume foods with the lowest phosphorus content. They were also educated to limit salt intake, preferring fresh foods, and promoting the use of spices and herbs to flavor dishes. In addition, all patients (independently of the group they belonged to) were given indications on the potassium content of different fruits and vegetables not to be too much restrictive in their consumption. Finally, both groups were given advice on cooking methods.

Normoprotein group was given the indication to try to eat the second dish only once a day or to split the portion of the second plate between lunch and dinner, if they wanted to keep the habit of making the meal complete. It was also given the indication to prefer, among protein sources, those of plant origin. We also indicated to alternate or replace cow's milk with plant substitutes such as: rice, almonds' or oats' drinks. Furthermore, we suggested to prefer white meat and to avoid offal and processed meat. Moreover, we indicated to substitute dried or smoked fish with fresh or frozen one.

LP group patients replaced pasta, bread, biscuits, etc., with LP substitutes. We allowed them to consume more animal products than NP, preferring white meat to red meat and trying to limit cold cuts as much as possible. Furthermore, they were advised to prefer fresh or frozen fish, instead of dried or smoked one as well as to prefer fresh cheeses to seasoned ones. As for legumes, we advised to combine them with bread or normal cereals, for protein complementarity.

Study design is represented in Supplementary Figure 1. None of the patients had ever been prescribed a LP diet or was followed by a nutritionist before randomization. To evaluate the metabolic effects of the two dietary regimens, we planned to leave other therapies unchanged unless there appeared conditions that could have endangered patients' safety during the time lag of the study.

The study had several endpoints.

Primary safety endpoints were:

- Intragroup MIS variations and intergroup MIS comparison at 6 months;
- Intergroup comparison of the number of patients that reached a MIS ≥ 8 at 6 months.
- Secondary endpoints consisted of intragroup and 6 months intergroup comparisons of:
- Renal function: cystatin-C-based eGFR;
- Metabolic complications of CKD: serum urea, serum phosphate and 24 h phosphaturia, intact parathormone (iPTH)
- fibroblast growth factor 23 (FGF23) (intact and C terminal), serum bicarbonate;
- Physical performance: short physical performance battery (SPPB) and handgrip strength;
- Inflammation: C reactive protein and interleukin 6 (IL-6);

Study outline is reported in Supplementary Figure 1.

Dietary compliance has been assessed by a trained nutritionist at months 1, 2, 3, and 6. Dietary consumption was estimated by using dietary diaries and normalized protein catabolic rate (nPCR) measurement at baseline, 3 and at 6 months. At months 1 and 2, dietary adherence was assessed using only dietary diaries.

Nutritional status and physical performance have been evaluated monthly for the first 3 months and then at 6 months.

Twenty-four hours urinary collection was started in the morning of the day preceding the visit and biochemical parameters were sampled the morning of the visits after an overnight fast of at least 12 h.

The study was conducted according to the ICP Good Clinical Practices Guidelines and it was approved by the Ethics Committee of our Institution (approval number 1274/2018). All patients had to sign an informed consent before participating in the study. The trial was registered on clinicaltrials.gov (ID NCT05015647).

### Body Composition and Nutritional Status

Anthropometric measurements included: body weight, height, body mass index (BMI), waist circumference (WC), mid-arm circumference (MAC), tricipital and bicipital skinfold thickness (TST, BST; measured with a Harpenden skinfold caliper). Mid Arm Muscle Circumference (MAMC) was calculated as follows: MAMC (cm) = MAC (cm) – (π × TST [cm]); these measurements were performed on the dominant arm as described elsewhere (20).

Nutritional status was assessed with malnutrition inflammation score (MIS) and by evaluating the presence of protein energy wasting (PEW).

Malnutrition-inflammation score is a validated scoring system for the assessment of malnutrition and inflammation syndrome in patients with CKD. MIS involves the evaluation of 10 different domains, each of which is categorized with 4 severity levels (score scale 0–3) (21). A total score of 4–7 was considered indicative of mild malnutrition and a score ≥8 of severe malnourishment (22).

PEW diagnosis was done using ISRNM criteria that fall into 4 distinct domains: serum chemistry, body mass, muscle mass, and dietary intake. Different indicators are proposed for each domain. For PEW diagnosis, it is sufficient for an indicator to be positive in at least 3 of the domains identified by the definition (23).

Protein intake was estimated by determining normalized protein catabolic rate (nPCR) on 24 h urinary urea excretion (24).

Dietary diaries were filled in the 3 days preceding the visits (Sunday to Tuesday) and we calculated nutrients intake by using the nutritional software Winfood (Medimatica Srl, Teramo Italy).

### Physical Performance and Muscle Strength

Physical performance was assessed by using SPPB, while muscle strength was assessed using handgrip strength.

SPPB includes: test of standing balance, 4-m walk, and time to rise from a chair five times (25). Each SPPB component test is scored from 0 to 4. Higher scores indicate better physical performance (26).

Handgrip strength was measured with Jamar dynamometer and was considered to be impaired for values <16 kg in women and <27 kg in men (27).

### Biochemical Parameters

All biochemical analyses for the evaluation of renal function, metabolic, and nutritional status were performed at the central laboratory of our Institution on the same days of the visits.

### Detection of Interleukin 6 Serum Levels, Cystatin C, and FGF23

Serum samples were frozen and stored at −80°C. Afterward, preserved samples were used for the quantitative determination of human IL-6, cystatin-C, and hepcidin. The determinations were performed with the following kits: Quantikine® HS ELISA anti Human IL-6 HS600C (R&D Systems, Space, Milano, Italy) with mean minimum detectable dose (MDD) of 0.031 pg/ml, Quantikine® ELISA Human Cystatin C Immunoassay (DSCTC0) with mean MDD of 0.102 ng/ml, and Quantikine® ELISA Human Hepcidin Immunoassay (DHP250) with mean MDD of 1.70 pg/ml. All tests were performed according to the instructions given by the manufacturer. Quantikine Immunoassay Control Group 8, 246, and QC220, respectively, for Cystatin C, IL-6, and Hepcidin (R&D Systems) were used to check the acceptability of the assays. Absorbance readings were measured at 450 nm by spectrophotometer (Xenius Safas, Monaco). All the values of cytokines were evaluated in duplicate. Both FGF23 (Intact and C-Term) plasma levels of patients were evaluated by commercially available ELISA Kit (Immuntopics Inc., CA, USA) with minimal detectable concentrations of 1.5 pg/ml and 1.5 RU/ml, respectively. Absorbance was read at a dual-wavelength of 450/630 nm. Replicate background measurements were subtracted to all 450 nm measures.

### Statistical Analysis

All data are expressed as mean ± SD or median ± IQR as appropriated. For inter-group comparison, we did ANCOVA test, moreover we did deltas follow-up baseline for each variable and compared them with the ANCOVA test, whereas intra-group comparison of parametric variables was done by using *t*-test for paired data, while intra-group comparison not parametric ones was done by using the Wilcoxon test. Proportions and categorical variables were compared by using the independent chi-squared (χ^2^) test. Regression analyses were performed by the Pearson's or Spearman's tests as appropriated. Statistical significance was set for *p* < 0.05. All analyses were carried out with Statview software version 5.0.1 and IBM SPSS software version 25.

## Results

### Population Characteristics

Characteristics of our population are summarized in Table 1. We enrolled at baseline 35 patients, the average age was 81 ± 6 years, 66% were men and 40% were diabetic, all these characteristics were well-balanced between the two groups of intervention. During the study, we had several dropouts. Eight patients left the study for the following reasons: 3 patients were started dialysis, 2 patients were died, and 3 patients were stopped the diet due to prolonged hospitalizations. The causes of dropouts were equally distributed between the two groups of intervention (Table 1).

### Nutritional Parameters, Physical Performance, and Muscle Strength

Table 2 describes the nutritional status of our patients at baseline and at 6 months (end of follow-up), subdivided by dietary prescription. At 6 months, NP had higher protein intake than LP group (0.81 ± 0.14 vs. 0.58 ± 0.15 g/kg/day, *p* < 0.05), which is not maintained in delta follow-up-baseline comparison. At 6 months, both groups increased their caloric intake with respect to baseline. Moreover, at the end of the study, both groups improved their MIS score. As regards to the nutritional status assessed with PEW, only LP group achieved a significant improvement from baseline to follow-up. No statistically significant differences were found for any Δ comparison.

**Table 2:** Nutritional status and nutritional intake at baseline vs. 6 months.

**Variables**	**NP** **baseline** **(*n* = 13)**	**LP** **baseline** **(*n* = 14)**	**NP** **6 months** **(*n* = 13)**	**LP** **6 months** **(*n* = 14)**	**Δ FU-BL** **NP** **(*n* = 13)**	**Δ FU-BL** **LP** **(*n* = 14)**	**Δ** **comparison** **NP-LP** ***p***
MIS	6 ± 2	6 ± 1.5	3 ± 2.5^a^	3 ± 3^a^	2 [−3; −0.5]	−2 [3; −0.75]	0.65
Nutritional status at MIS							
Risk of malnutrition, *n*	13	14	6	5	n.a.	n.a.	
Well-nourished, *n*	0	0	7	9	n.a.	n.a.	
Malnourished, *n*	0	0	0	0	n.a.	n.a.	
PEW, *n*	6	6	5	4^a^	n.a.	n.a.	
Nutritional parameters							
Albumin (g/dl)	3.7 [3.5; 4.1]	3.7 [3.6; 4]	3.8 [3.6; 4.1]	3.9 [3.7; 4.1]	−0.02 [−0.17; 0.24]	0.09 [−0.04; 0.25]	0.60
Prealbumin (mg/dl)	29 [21.5; 32]	29.5 [24.5; 31]	26.0 [21.2; 30]	26.5 (22, 28)	−2 [−6; 3]	−1.5 [−5; 2.25]	0.44
Total cholesterol (mg/dl)	144 [125; 213]	155 [133; 175]	130 [108; 170]	155 [122; 170]	−7 [−38; 7.5]	1 [−17; 4.5]	0.33
Transferrin (mg/dl)	224 ± 50	203 ± 35	228 ± 46^a^	217 ± 40	17 [−6; 29.5]	8 [−30.2; 33.25]	0.68
Lymphocytes (n/mm^3^)	1,428 ± 690	1,942 ± 1,216	1,497 ± 448	1,598± ,548	1.3 [−2; 4.2]	−2.1 [−7.4; 2]	0.05
Proteins and calories intake							
nPCR (g/kg/24 h)	0.68 ± 0.18	0.66 ± 0.13	0.81 ± 0.14^c^	0.58 ± 0.15^c^	0.149 ± 0.122	0.075 ± 0.298	0.38
Kcal/kg	18 (14, 18)	19 (15, 25)	28 (22, 30)^b^	29 [23.7; 32]^b^	10.9 [2.9; 15.2]	9 [3.3; 11.3]	0.70

*MIS, Malnutrition Inflammation Score; PEW, protein energy wasting*.

a *<0.05 baseline vs. follow-up*.

b *<0.01 baseline vs. follow-up*.

c *<0.05 vs. 6 months*.

Table 3 shows the comparison between the two intervention groups regarding body composition, physical performance, and muscle strength evaluated at baseline and follow-up. No statistically significant differences were found for any of the variables considered at both intergroup, intragroup, and deltas intergroup comparison.

**Table 3:** Body composition, physical performance, and muscle strength (baseline vs. 6 months).

**Variables**	**NP** **baseline** **(*n* = 13)**	**LP** **baseline** **(*n* = 14)**	**NP** **6 months** **(*n* = 13)**	**LP** **6 months** **(*n* = 14)**	**Δ FU-BL** **NP** **(*n* = 13)**	**Δ FU-BL** **LP** **(*n* = 14)**	**Δ** **comparison** **NP-LP** ***p***
Weight (Kg)	73.5 [68; 88]	73.5 [63; 83]	71.8 [67.6; 87]	75 [59.5; 83]	−0.5 [−3; 2.2]	−0.25 [−3.1; 1.6]	0.92
BMI (kg/m^2^)	27.8 ± 6.6	26.3 ± 6.6	27.0 ± 6.3	27.8 ± 6.5	−0.2 [−1.3; 1.2]	−0.3 [1.3; 0.6]	0.52
waist circumference (cm)	104 [93.5; 109]	103 [95; 113.7]	101 [91.5; 106.5]	101 [96; 110]	1 [−4; 3]	0.25 [−1.5; 2.6]	0.63
MAC (cm)	29 [20.4; 26.1]	30.3 [21.5; 28.7]	30 [21.8; 26.6]	30 [23.2; 28]	0 [−1.63; 1.7]	0.5 [−1.4; 2.2]	0.81
TST (mm)	14.5 [11; 19.6]	14.3 [12; 18.9]	14.8 (12, 17)	12.4 (10, 17)	−0.25 [−3.3; 2.8]	−2.1 [−5; 0.5]	0.86
BST (mm)	9 [7.8; 13.5]	9.75 [7.8; 12.5]	10 [7.2; 14]	9.5 [5.5; 12]	−0.2 [−6.7; 2]	−2.8 [−5; 1.8]	0.49
MAMC (cm)	24.3 [20.4; 26.1]	25.2 [21.5; 28.7]	24.2 [21.8; 26.6]	24.3 (21, 26)	0 [−1.6; 1.7]	0.5 [−1.4; 2.2]	0.94
Physical Performance and muscle strength							
SPPB	7 [6.5; 9.5]	8 (6, 10)	8 (6, 10)	9 (7, 10)	0.23 ± 1.8	0.86 ± 1.9	0.4
Handgrip strength (Kg)	22 [16; 28.5]	27 [18.7; 30.2]	20 [15.5; 25]	28 (19, 29)	−0.62 ± 3.2	1 ± 4.7	0.31
Handgrip strength reduction *n* (%)	4(31)	3(21)	5(38)	3(21)	n.a.	n.a	n.a

*BMI, body mass index, SPPB, short physical performance battery*.

### Kidney Function and Other Metabolic Parameters

Kidney function and other metabolic parameters of our patients are reported in Table 4. At 6 months, patients on LP diet had higher eGFR (estimated by using cystatin c) and lower serum urea concentration than NP group. eGFR (estimated by plasma creatinine), as well as creatinine clearance, did not show any difference at both intragroup and intergroup comparison.

**Table 4:** Kidney function and metabolic parameters (baseline vs. 6 months).

**Variables**	**NP** **baseline** **(*n* = 13)**	**LP ** **baseline** **(*n* = 14)**	**NP** **6 months** **(*n* = 13)**	**LP** 6 months **(*n* = 14)**	**Δ FU-BL** **NP** **(*n* = 13)**	**Δ FU-BL** **LP** **(*n* = 14)**	**Δ** **comparison** **NP-LP** ***p***
eGFR Cys C (ml/min/1.73 m^2^)	14 ± 4	17 ± 7	12 ± 4^a^	17 ± 6^a^	−2 [−6.5; 2]	1 [−5.7; 4]	0.68
eGFR creat (ml/min/1.73 m^2^)	19 ± 6	19 ± 7	19 ± 9	18.5 ± 7	−2 [−4.5; 2.5]	−1.5 [−3; 1.5]	0.46
Creatinine Clearance (ml/min)	19 (15, 23)	17 (16, 20)	18 [14.5; 26]	20 (13, 19)	−1.1 [−5; 1.7]	−1.5 [−2.8; 1.5]	0.30
Serum Urea (mg/dl)	124 ± 32	113 ± 26	138 ± 30^c^	105 ± 65^c^	17.7 ± 40.1	8.6 ± 23.4	0.47
Urinary sodium (mmol/24 h)	91 [81; 182]	141 [78; 178]	84 [75; 152]	116 [96; 136]	−8 [−40; 20]	−22.5 [−60.2; 35]	0.89
HCO_3_ (mEq/L)	26.4 ± 6.2	26.6 ± 5.5	24.8 ± 5.9	24.7 ± 5.5	−0.76 ± 2.9	−0.44 ± 3	0.77

*eGFR, estimated glomerular filtration rate; HCO3, bicarbonate. a <0.05 vs. 6 months*.

Table 5 shows the variables related to calcium-phosphorus metabolism. At 6 months, patients in LP group had lower parathormone (PTH) levels than NP group [68 (58.5; 71) vs. 99 (80; 141), *p* < 0.05], which is also maintained in delta follow-up-baseline comparison [−3.2 (−22.3; 15.3) vs. 18.2 (3.5; 44), *p* = 0.049]. FGF23 (intact and C-terminal) increased in both LP and NP groups between baseline and 6 months but not in Δ comparison.

**Table 5:** Assessment of calcium-phosphorous metabolism.

**Variables**	**NP** **baseline** **(*n* = 13)**	**LP ** **baseline** **(*n* = 14)**	**NP** **6 months** **(*n* = 13)**	**LP** **6 months** **(*n* = 14)**	**Δ FU-BL** **NP** **(*n* = 13)**	**Δ FU-BL** **LP** **(*n* = 14)**	**Δ** **comparison** **NP-LP** ***p***
FGF-23 intact (pg/mL)	86 [57; 194]	70 [48; 98]	143 [119; 186]^a^	126 [90; 410]^a^	65.1 [−9.7; 155.3]	63.2 [38; 235.2]	0.48
FGF-23 c-terminal (RU/mL)	142 [56.6; 175]	77 [30.3; 112]	157 [76.7; 281]^a^	111 [63; 384]^a^	28.2 [−55.8; 140.1]	67.2 [−22.5; 124.7]	0.40
Intact/c-terminal	0.98 [0.5; 1.4]	2.2 [0.6; 2.5]	1.1 [0.8; 1.3]	1.3 [0.9; 1.4]	−0.09 [−0.4; 0.3]	0.3 [−1.4; 0.6]	0.6
25OH-vitamin D (ng/ml)	24 (16, 26)	24 [18; 35.5]	25 (16, 32)	30 (22, 32)	−3.3 [−7.4; 11.4]	1.9 [−0.6; 13.9]	0.25
1,25OH-vitamin D (pg/mL)	27 [17.6; 33.5]	26 (19, 29)	28 (18, 28)	30 [23.5; 39]	3.6 [−3.5; 11.9]	−0.25 [−2.3; 6.1]	0.61
Phosphorous (mg/dl)	3.6 ± 0.8	3.8 ± 0.8	3.7 ± 0.5	3.9 ± 0.9	−0.1 [−0.4; 0.3]	−0.1 [−0.3; 0.4]	0.63
Urinary phosphorous (mg/24 h)	400 ± 145	520 ± 210	340 ± 108	400 ± 140	−50 [−200; 25]	−65 [−155; 45]	0.72
PTH (ng/L)	80 [66; 118]	65 [55.5; 80]	99 [80; 141]^b^	68 [58.5; 71]^b^	18.2 [3.5; 44]	−3.2 [−22.3; 15.3]	0.049

*FGF 23, fibroblast growth factor 23; PTH, parathormone*.

a *<0.01 baseline vs. follow-up*.

b *<0.05 vs. 6 months*.

### Assessment of Inflammatory Status

Table 6 describes the variables related to inflammatory status, subdivided into the two treatment groups at baseline and at 6 months. We did not observe any difference at both intra and intergroup analysis.

**Table 6:** Assessment of inflammatory status.

**Variables**	**NP** **baseline** **(*n* = 13)**	**LP ** **baseline** **(*n* = 14)**	**NP** **6 months** **(*n* = 13)**	**LP** **6 months** **(*n* = 14)**	**Δ FU-BL** **NP** **(*n* = 13)**	**Δ FU-BL** **LP** **(*n* = 14)**	**Δ** comparison NP-LP ***p***
IL-6 (pg/mL)	11.7 (6, 16)	6.7 (5, 15)	8.9 (6, 8)	7.9 (7, 17)	0.05 [−2.9; 1.7]	0 [−3.2; 9.6]	0.16
CRP (mg/dl)	0.20 [0.08; 0.9]	0.30 [0.12; 0.55]	0.28 [0.13; 1.07]	0.73 [0.24; 1.41]	0.003 ± 0.83	0.065 ± 1.5	0.18

*IL-6, Interleukin 6; CRP, C-reactive protein*.

## Discussion

The aim of this pilot study was to assess whether in older patients with CKD at risk of malnutrition LP diet could be as safe as NP diet. The primary endpoint was to assess whether there were differences between LP and NP group regarding the development of malnutrition (by comparing MIS score or the onset of PEW in two groups). At the end of the study, none of patients included in the two treatment groups developed malnutrition. Noteworthy, at the end of the study, patients treated with both LP and NP diets achieved an improvement of their nutritional status (LP: MIS from 6 ± 1.5 to 3 ± 3, *p* = 0.0063; NP: MIS 6 ± 2 to 3 ± 2.5, *p* = 0.0117). Furthermore, patients in LP group showed a significant reduction of PEW (6 vs. 4, *p* = 0.04). Therefore, the primary result of our study is that in older patients with CKD at risk of malnutrition, the adoption of LP diet supplemented with protein-free foods does not induce malnutrition. This finding seems to counteract current indications established for older individuals at the risk of malnutrition in the general population that suggest adopting normocalorie and normo/high protein diets (14, 28–30). This could, however, be liable to multiple interpretative keys. One of these could be that the use of protein-free products may help to maintain an adequate energy intake excluding, or at any rate reducing, the consumption of cereals and derivatives and allowing in proportion a greater supply of high biological value proteins. In patients with CKD, this dietary approach may allow to limit the accumulation of derivatives of protein metabolism without increasing the risk of nutritional inadequacy (5).

We did not find any significant variation of nutritional biochemical markers (albumin, prealbumin, transferrin, cholesterol, and lymphocyte count) in both inter and intragroup comparisons. This seems to confirm that none of the two diets does adversely affect the nutritional status in the medium term. However, it has to be recognized that conditions such as changes in inflammatory state, progressive decline in kidney function, specific pharmacological treatments, and changes in blood iron level could help to limit the sensitivity and specificity of these parameters in defining the nutritional status of these patients (5). In our analysis, we have specifically addressed the variations of inflammatory status (CRP and IL6) in the two groups without finding significant differences. We concluded the assessment of nutritional status by evaluating muscular strength (handgrip strength test) and physical performance (SPPB). However, we did not find any difference in both these parameters either by comparing the groups to each other nor within the single group by comparing the different timepoints.

The secondary objectives of the study were manifold. In fact, we assessed whether, in these patients, LP diet could have had a specific indication to slow down the progression of kidney disease and to delay the onset of its metabolic complications (as urea accumulation, metabolic acidosis, and alterations of calcium-phosphorus metabolism).

When eGFR was estimated by using CKD-EPI_cr_, we did not find any difference between LP and NP patients. However, when we used CKD-EPI_cys_, at the 6th month, eGFR was higher in LP than in NP patients (respectively: 17 ± 6 vs. 12 ± 4 ml/min/1.73 m^2^; *p* < 0.05). Which is the best method to estimate the eGFR in older patients is still debated. In a recent paper, Torregiani and co-authors (31) found that the most frequently used formulae based on the creatinine values had similar performances in classifying the stage of CKD independently of patients' age. In the general CKD population (32), the formulae that contain both creatinine and cystatin C values are more precise and accurate to estimate GFR with respect to those that are based solely on creatinine. These results are confirmed also in older patients with CKD (33). However, creatinine and cystatin C are differently influenced by factors as: age, comorbidities, dietary protein intake, muscular mass, and inflammation (34). In particular, it has to be considered that, while serum creatinine concentration is reduced by LP diet and reduced muscular mass, these variables do not affect serum cystatin C and its related formulae (35). Therefore, it is plausible that the different results of creatinine and cystatin C eGFR that were found between NP and LP group at the 6th month may depend on the lower protein intake of LP group. Therefore, it is plausible that LP diet may be more indicated than NP diet to preserve renal function in older patients with advanced CKD at the risk of malnutrition. However, although the absolute value of cystatin C eGFR was statistically significant, the comparison between the deltas was not. It is possible that the follow-up period was too short since the cohort was already selected to have a stable renal function over time.

At the end of the study, LP patients had significantly lower levels of urea (105 ± 65 vs. 138 ± 30, *p* < 0.05) and lower PTH values [68 (58.5; 71) vs. 99 (80; 141) ng/L, *p* < 0.05] than NP. We did not find any difference of serum and 24 h urinary phosphate, as well as of bicarbonate concentration in both group of treatment at none of the timepoints. These differences were independent of drugs and supplements that may have influenced those variables (Supplementary Table 11). In patients with CKD, secondary hyperparathyroidism has multifactorial etiology (36). From a nutritional point of view, the main factors which could have influenced changes in PTH are the variations of phosphorus intake and phosphoremia, where latter is in turn regulated by Vitamin D, PTH, and FGF23. In both groups of intervention, phosphoremia as well as phosphaturia fell within the normal range without differences either between or within groups. Likely, FGF23 levels were comparable in the two groups of treatment although they increased from baseline to the end of the study in both groups. Association between the variables related to calcium-phosphorus metabolism is reported in Supplementary Tables 9, 10.

Patients with advanced CKD are at increased risk of malnutrition due to CKD itself; however, in this study, we specifically assessed the safety of LP diet in older patients with advanced CKD that were at the risk of malnutrition at MIS and presented a lack of appetite and a spontaneous reduction of proteins and energy intake. Current guidelines (14, 28–30) suggest to avoid the prescription of LP diets in malnourished patients with CKD. However, these same guidelines do not specify which nutritional interventions could be indicated for older patients with CKD at risk of malnutrition (16). Consequently, the most widely adopted indication is that of prescribing a normocalories (30 kcal/kg) and NP (0.8 g/kg) diet as it is suggested for the general elderly population (37–39). However, in patients with advanced CKD, an increase in protein intake could lead to an increase in urea and phosphates as well as a reduction in bicarbonate (6). These are all variations that contribute to generate the metabolic imbalance that characterizes uremic syndrome. In our study, patients on the LP diet had significantly lower levels of urea than the NP group. However, we did not find significant variations in serum and urinary phosphate among patients undergoing the two different dietary regimes. These results could have been influenced by the different proportions of proteins of animal and plant origin prescribed to the two treatment groups. Indeed, patients on LP diet were prescribed to consume a higher proportion of animal proteins to ensure the intake of essential amino acids. While patients belonging to the NP group were prescribed to increase the consumption of proteins of plant origin to contain phosphate intake (Supplementary Table 7).

Although the results are promising, our study includes a limited number of patients, thus its results should be considered with some cautions. However, we have to consider that the purpose of a pilot study is to test the methodology planned to verify a hypothesis. On this regard, we believe that we confirmed the goodness of our methods. Furthermore, we believe that our results highlighted some critical issues to be considered for future studies:

- To ensure patients' adherence to the diet, it would be better to plan monthly check-ups comprehensive of the assessment of protein (by estimating the nPCR) and caloric intakes (by fulfilling the dietary diaries).
- It would be advisable to define a priori the proportional distribution between animal and vegetable proteins in LP and NP treatment groups.
- The difference in protein intake between both groups is small. However, we compared for the first time, the nutritional safety of different protein intakes indicated by contrasting guidelines and expert consensus (40, 41) in this population.
- The follow-up of 6 months may be too short to evaluate changes in eGFR. However, regarding nutritional status that was the main aim of our study, this time can be considered suitable to have reliable results.
- Future studies should evaluate whether prescribing caloric-protein or exclusively caloric supplements to facilitate the achievement of individual caloric and/or protein quota when patients are unable to reach it spontaneously.

Finally, another limitation of our study was that as MIS and ISRNM definitions of PEW are subjective assessment of nutritional status, the fact that the study did not have a blind design may have affected the clinical judgment of the operator.

In conclusion, the most relevant result of our study is that in older patients with advanced CKD at risk of malnutrition, the prescription of a LP diet does not induce malnutrition. Conversely, our results demonstrate that in these patients, the prescription of LP and normocaloric diet allows to improve the nutritional status as much as NP diet. Moreover, LP diet seems to have greater benefits than the NP with respect to the progression of kidney disease and the onset of its metabolic complications. Therefore, our results seem to confirm what has been hypothesized in some previous studies conducted in patients with CKD not at the risk of malnutrition (12, 15, 17), namely that the most important aspect for the prevention of malnutrition in patients with CKD is to maintain adequate calorie consumption rather than prescribing an increase of protein intake.

## Data Availability Statement

The datasets presented in this study can be found in online repositories. The names of the repository/repositories and accession number(s) can be found below: https://osf.io/ehjpb/files/, https://osf.io/ehjpb/files/.

## Ethics Statement

The studies involving human participants were reviewed and approved by Ethics Committee of Fondazione IRCCS Ca' Granda Ospedale Maggiore Policlinico di Milano. The patients/participants provided their written informed consent to participate in this study.

## Author Contributions

SV and LC: conceptualization, methodology, and writing—original draft preparation. LC and PMo: software. SV and PMe: validation. LC, SV, SA, DM, MI, and CA: formal analysis. LC: investigation. PMe: resources and funding acquisition. LC, SA, and DM: data curation. PMe and GC: writing—review and editing and supervision. SV: visualization and project administration. All authors contributed to the article and approved the submitted version.

## Conflict of Interest

The authors declare that the research was conducted in the absence of any commercial or financial relationships that could be construed as a potential conflict of interest.

## Publisher's Note

All claims expressed in this article are solely those of the authors and do not necessarily represent those of their affiliated organizations, or those of the publisher, the editors and the reviewers. Any product that may be evaluated in this article, or claim that may be made by its manufacturer, is not guaranteed or endorsed by the publisher.
