# Supplementary material for: Possible Benefits of a Low Protein Diet in Older Patients With CKD at Risk of Malnutrition: A Pilot Randomized Controlled Trial
Source: Front Nutr. 2022 Jan 26;8:782499. doi: 10.3389/fnut.2021.782499 (PMC8860492; doi:10.3389/fnut.2021.782499)
Supplement: Supplementary file 1 [file Table_1.DOC]

**
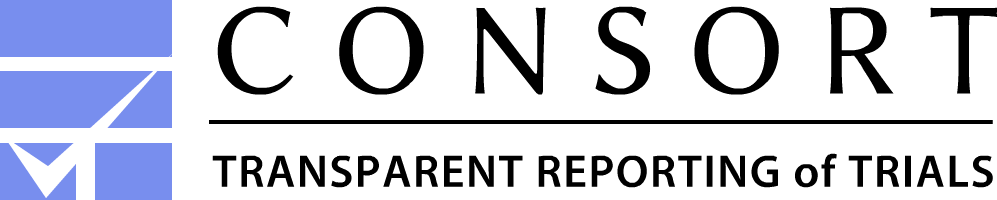
**

**CONSORT 2010 Flow Diagram**

**Allocation**

**Analysis**

**Follow-Up**

**Enrollment**

Assessed for eligibility (n=50)

Excluded (n= 15)

♦  Not meeting inclusion criteria (n=14)

♦  Declined to participate (n= 1)

♦  Other reasons (n= 0)

Analysed (n=13)
♦ Excluded from analysis (give reasons) (n=0)

Lost to follow-up (give reasons) (n= 4): 2 started dialysis, 2 for prolonged hospitalization)

Discontinued intervention (give reasons) (n=0)

Allocated to intervention LP (n= 17)

♦ Received allocated intervention (n= 17)

♦ Did not receive allocated intervention (give reasons) (n= 0)

Lost to follow-up (give reasons) (n= 4): 1 started dialysis, 2 died, 1 for prolonged hospitalization

Discontinued intervention (give reasons) (n=0)

Allocated to intervention NP (n= 18)

♦ Received allocated intervention (n=18)

♦ Did not receive allocated intervention (give reasons) (n= 0)

Analysed (n=14)
♦ Excluded from analysis (give reasons) (n=0)

Randomized (n= 35)
