# Supplementary material for: Possible Benefits of a Low Protein Diet in Older Patients With CKD at Risk of Malnutrition: A Pilot Randomized Controlled Trial
Source: Front Nutr. 2022 Jan 26;8:782499. doi: 10.3389/fnut.2021.782499 (PMC8860492; doi:10.3389/fnut.2021.782499)
Supplement: Supplementary file 2 [file Data_Sheet_1.docx]

Supplementary Material

**Figure 1.** Study outline


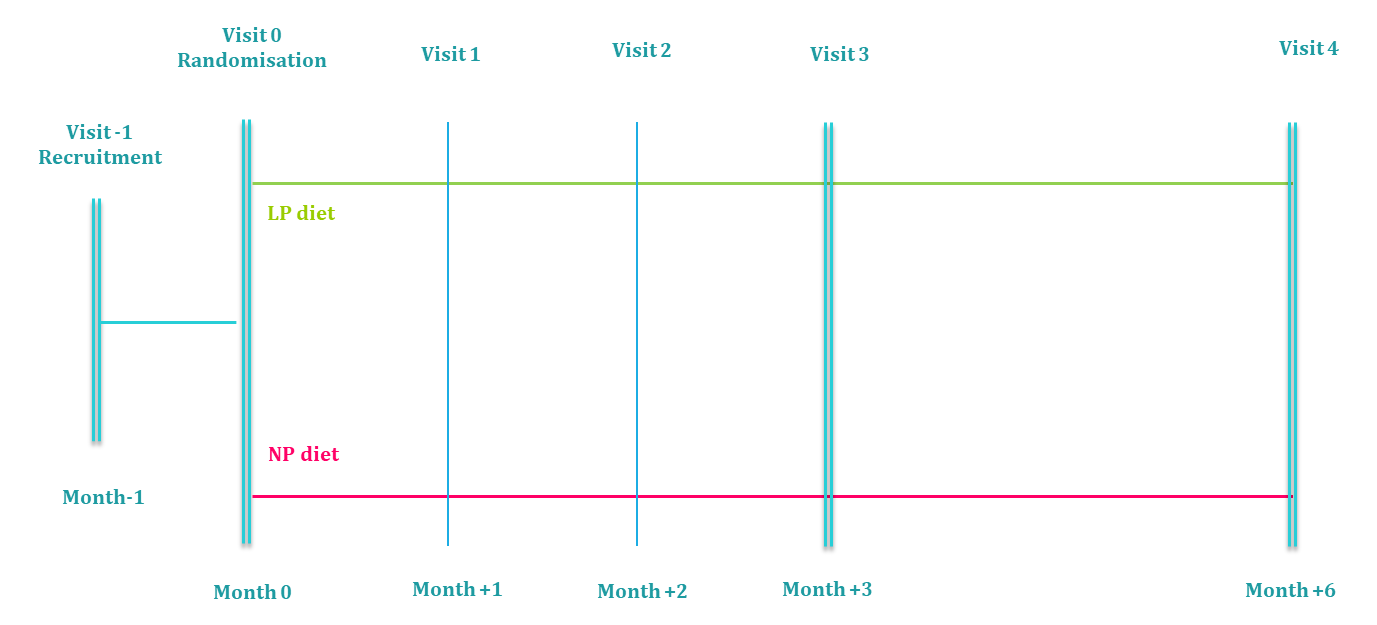


**Table 7.** Dietary habits derived by diaries

| **Variables** | **NP**  **Baseline**  (n=13) | **LP**  **Baseline**  (n=14) | **NP**  **3 months**  (n=13) | **LP**  **3 months**  (n=14) | **NP**  **6 months**  (n=13) | **LP**  **6 months**  (n=14) | **p**  **baseline** | | **p**  **3 months** | | **p**  **6 months** |
| --- | --- | --- | --- | --- | --- | --- | --- | --- | --- | --- | --- |
| Carbohydrates, % | 46 | 52 | 54 | 58 | 53 | 57 | | 0.57 | | 0.45 | 0.59 |
| Lipids, % | 38 | 36 | 32 | 32 | 36 | 33 | | 0.47 | | 0.41 | 0.38 |
| Animal proteins, % | 60 | 67 | 43 | 77 | 41 | 75 | | 0.42 | | **0.04** | **0.048** |
| Vegetal proteins, % | 40 | 33 | 57 | 23 | 59 | 25 | | 0.72 | | **0.041** | **0.037** |

*p refers to comparisons between NP and LP at any of the timepoints*

**Table 8.** Comparison of nutritional and body composition variables (baseline-3 months)

| **Variables** | **LP**  **Baseline**  (n=14) | **NP**  **3 months**  (n=13) | **LP**  **3 months**  (n=14) | **Δ FU-BL**  **NP**  (n=13) | **Δ FU-BL**  **LP**  (n=14) | **Δ comparison**  **NP-LP**  **p** |
| --- | --- | --- | --- | --- | --- | --- |
| Urinary urea 24h (mg/24h) | 12800±4762 | 15560±5392 | 14280±4380 | -720[-2380;2396] | 1560[-1150;4215] | 0.55 |
| nPCR (g/kg/24h) | 0.66±0.13 | 0.80±0.2 | 0.67±0.2 | -0.042[-0.18;0.11] | 0.12[-0.063;0.24] | 0.87 |
| Kcal/Kg | 19±4 | 31.1±3.2 | 33.1±3.2 | 12.7[6.6;15.7] | 10.1[6.4;17.8] | 0.74 |

|  |  |  |  |  |  | |
| --- | --- | --- | --- | --- | --- | --- |
|  |  |  |  |  |  |  |
|  |  |  |  |  |  |  |
|  |  |  |  |  |  |  |

nPCR, normalized Protein catabolic rate;

**Table 9.** Correlations between FGF23 (intact and C-terminal) and other factors determining the calcium-phosphoric balance at baseline

| **Variables** | **Overall cohort**  **(n=27)** | | **NP**  **BL**  **(n=13)** | | **LP**  **BL**  **(n=14)** | |
| --- | --- | --- | --- | --- | --- | --- |
| **FGF-23 intact** | **r** | **p** | **r** | **p** | **r** | **p** |
| Phosphorus (mg/dl) | **0.39** | **0.021** | 0.33 | 0.19 | 0.47 | 0.059 |
| Vitamin D 25OH (ng/ml) | -0.12 | 0.49 | -0.13 | 0.63 | -0.17 | 0.50 |
| Vitamin D 1,25 OH (pg/mL) | **-0.50** | **0.0038** | **-0.53** | **0.041** | **-0.54** | **0.024** |
| Creatinine clearance (mL/min) | **-0.44** | **0.0098** | **-0.57** | **0.016** | **-0.28** | **0.27** |
| Urinary Phosphorus 24h (mg/24h) | -0.18 | 0.30 | -0.077 | 0.77 | -0.25 | 0.32 |
| Phosphorus Intake (mg) | 0.11 | 0.57 | 0.073 | 0.80 | 0.21 | 0.42 |
| **FGF-23 c-terminal** | | | | | | |
| Phosphorus (mg/dl) | 0.18 | 0.30 | 0.35 | 0.16 | 0.018 | 0.94 |
| Vitamin D 25OH (ng/ml) | -0.058 | 0.75 | -0.037 | 0.89 | -0.14 | 0.59 |
| Vitamin D 1,25 OH (pg/mL) | -0.17 | 0.36 | -0.29 | 0.28 | -0.14 | 0.60 |
| Creatinine clearance (mL/min) | -0.160 | 0.37 | -0.34 | 0.17 | -0.002 | 0.99 |
| Urinary Phosphorus 24h (mg/24h) | 0.11 | 0.54 | 0.18 | 0.51 | 0.19 | 0.47 |
| Phosphorus Intake (mg) | 0.17 | 0.36 | 0.20 | 0.49 | 0.34 | 0.19 |

**Table 10.** Correlations between FGF23 (intact and C-terminal) and other factors determining calcium-phosphoric balance at the end of the study

| **Variables** | **Overall cohort**  **(n=27)** | | **NP**  **6 months**  **(n=13)** | | **LP**  **6 months**  **(n=14)** | |
| --- | --- | --- | --- | --- | --- | --- |
| **FGF-23 intact** | **r** | **p** | **r** | **p** | **r** | **p** |
| Phosphorus (mg/dl) | **0.82** | **<0.0001** | **0.86** | **0.0007** | **0.60** | **0.0019** |
| Vitamin D 25OH (ng/ml) | 0.34 | 0.10 | 0.36 | 0.27 | 0.43 | 0.15 |
| Vitamin D 1,25 OH (pg/mL) | -0.11 | 0.63 | 0.25 | 0.46 | -0.45 | 0.14 |
| Creatinine clearance (mL/min) | **-0.50** | **0.0123** | -0.49 | 0.13 | **-0.59** | **0.035** |
| Urinary Phosphorus 24h (mg/24h) | 0.23 | 0.28 | 0.42 | 0.19 | 0.27 | 0.37 |
| Phosphorus Intake (mg) | 0.001 | 0.99 | -0.21 | 0.54 | 0.32 | 0.34 |
| **FGF-23 c-terminal** | | | | | | |
| Phosphorus (mg/dl) | 0.24 | 0.25 | 0.33 | 0.31 | 0.093 | 0.75 |
| Vitamin D 25OH (ng/ml) | 0.20 | 0.33 | -0.017 | 0.96 | **0.63** | **0.013** |
| Vitamin D 1,25 OH (pg/mL) | -0.11 | 0.62 | 0.093 | 0.79 | -0.25 | 0.41 |
| Creatinine clearance (mL/min) | -0.050 | 0.81 | -0.044 | 0.89 | -0.007 | 0.98 |
| Urinary Phosphorus 24h (mg/24h) | 0.37 | 0.065 | 0.37 | 0.26 | **0.61** | **0.021** |
| Phosphorus Intake (mg) | 0.32 | 0.14 | 0.18 | 0.60 | 0.51 | 0.10 |

**Table 11.** Drug distribution in patients who completed the study

| **Variables** | **Overall cohort**  **baseline**  (n=27) | **NP**  **baseline**  (n=13) | **LP**  **baseline**  (n=14) | **NP**  **6 months**  (n=13) | **LP**  **6 months**  (n=14) | **NP LP**  **p baseline vs 6 months** | |
| --- | --- | --- | --- | --- | --- | --- | --- |
| RAS- inhibitor, n | 14 | 7 | 7 | 7 | 8 | ns | ns |
| Diuretic, n | 16 | 8 | 8 | 8 | 8 | ns | ns |
| Calcifediol, n | 21 | 11 | 10 | 11 | 11 | ns | ns |
| Calcitriol, n | 15 | 6 | 9 | 7 | 9 | ns | ns |
| Phosphate binder, n | 0 | 0 | 0 | 0 | 0 | - | - |
| Bicarbonate, n | 10 | 6 | 4 | 5 | 4 | ns | ns |

**Table 12.** Nutritional status at MIS and PEW at last known time point.

| **Variables** | **NP**  **baseline**  (n=18) | **LP**  **baseline**  (n=17) | **NP**  **End of study**  (n=18) | **LP**  **End of study**  (n=17) | **NP LP**  **p baseline vs end of study** | |
| --- | --- | --- | --- | --- | --- | --- |
| MIS | 6±2 | 6±1.5 | 4.5±3 | 3±3.3 | 0.0082 | 0.0028 |
| *Nutritional status at MIS* |  |  |  |  |  |  |
| Risk of malnutrition, n | 18 | 17 | 10 | 8 | 0.25 | 0.33 |
| Well nourished, n | 0 | 0 | 9 | 10 | 0.25 | 0.27 |
| Malnourished, n | 0 | 0 | 0 | 0 |  |  |
| PEW, n | 9 | 8 | 8 | 6 | 0.40 | 0.058 |

MIS, Malnutrition Inflammation Score; PEW, protein energy wasting

**Table 13.** Comparison of metabolic components (baseline-3 months)

| **Variables** | **NP**  **Baseline**  (n=13) | **LP**  **Baseline**  (n=14) | **NP**  **3 months**  (n=13) | **LP**  **3 months**  (n=14) | **Δ FU-BL**  **NP**  (n=13) | **Δ FU-BL**  **LP**  (n=14) | **Δ comparison**  **NP-LP**  **p** |
| --- | --- | --- | --- | --- | --- | --- | --- |
| eGFR creat (ml/min/1,73m2) | 19±6 | 19±7 | 19±6 | 20±7.5 | 0.5[-2;2] | -1[-1.2;0] | 0.68 |
| Serum Urea (mg/dl) | 124±32 | 113±26 | 116±45 | 100±46 | -5.1±25.2 | 0.52±35 | 0.64 |
| Urinary sodium (mmol/24h) | 91±98 | 141±89 | 106±59 | 122±63 | -28.5[-70;32] | 6[-10.5;27.7] | **0.035** |
| HCO3 (mEq/L) | 26.4±6.2 | 26.6±5.5 | 26.2±4.7 | 26.3±3.2 | -0.1[-1.4;2] | 0.25[-1.6;2.4] | 0.65 |
| Phosphorous (mg/dl) | 3.6±0.8 | 3.8±0.8 | 3.8±0.8 | 3.6±1.0 | -0.05[-0.4;0.3] | -0.05[-0.3;0.4] | 0.78 |
| PTH (ng/L) | 80±49 | 65±17 | 87±59^c^ | 60±16^c^ | -0.1[-12.8;8.8] | 0[-32.3;20] | 0.74 |

|  |  |  |  |  |  |
| --- | --- | --- | --- | --- | --- |
|  |  |  |  |  |  |
|  |  |  |  |  |  |
|  |  |  |  |  |  |
|  |  |  |  |  |  |
|  |  |  |  |  |  |
|  |  |  |  |  |  |

eGFR creat, estimated Glomerular Filtration Rate calculated using creatinine; HCO3, bicarbonate; PTH, parathormone.

^a^<0.05 baseline vs follow-up

^b^<0.01 baseline vs follow-up

^c^<0.05 versus 6 months
